# Genome-Wide and Transcriptome Analysis of Jacalin-Related Lectin Genes in Barley and the Functional Characterization of *HvHorcH* in Low-Nitrogen Tolerance in Arabidopsis

**DOI:** 10.3390/ijms242316641

**Published:** 2023-11-23

**Authors:** Xiaoyan Quan, Chen Meng, Chunjuan Xie, Huifang Sun, Boyang Xu, Ramon Santos Bermudez, Wenxing He

**Affiliations:** School of Biological Science and Technology, University of Jinan, Jinan 250022, China

**Keywords:** genome-wide analysis, JRL, barley, low-nitrogen stress, transcriptome

## Abstract

The jacalin-related lectins (JRLs) are widely distributed in plants and are involved in plant development and multiple stress responses. However, the characteristics of the *HvJRL* gene family at the genome-wide level and the roles of JRLs in barley’s response to low-nitrogen (LN) stress have been rarely reported. In this study, 32 *HvJRL* genes were identified and unevenly distributed at both ends of the seven chromosomes in barley. HvJRL proteins generally exhibited low sequence similarity but shared conserved jacalin domains by multiple sequence analysis. These proteins were classified into seven subfamilies based on phylogenetic analysis, with a similar gene structure and conserved motifs in the same subfamily. The *HvJRL* promoters contained a large number of diverse *cis*-elements associated with hormonal response and stress regulation. Based on the phylogenetic relationships and functionally known JRL homologs, it was predicted that some *HvJRLs* have the potential to serve functions in multiple stress responses but not nutrition deficiency stress. Subsequently, nine differentially expressed genes (DEGs) encoding eight HvJRL proteins were identified in two barley genotypes with different LN tolerance by transcriptome analysis. Furthermore, *35S:HvHorcH* transgenic Arabidopsis seedlings did enhance LN tolerance, which indicated that *HvHorcH* may be an important regulator of LN stress response (LNSR). The *HvJRL* DEGs identified herein could provide new candidate genes for LN tolerance studies.

## 1. Introduction

Plant lectins are a kind of carbohydrate-binding protein with diverse functional roles in plants [1,2]. Jacalin-related lectins (JRLs) are a new subfamily of plant lectins with at least one jacalin domain [3]. According to the specificity of glycosylation binding, JRLs can be divided into two types, i.e., galactose-specific JRLs (gJRLs) and mannose-specific JRLs (mJRLs) [4,5]. So far, the number of known mJRLs is significantly higher than that of gJRLs. Based on their domain type, JRLs can be grouped into three categories: merojacalins with only one single jacalin domain; holojacalins with multiple jacalin domains; and chimeric jacalins with jacalin domain(s) and other domain(s), such as dirigent, NB-ARC, and Pkinase domains [2,6]. The expansion of the jacalin domain and their fusion with other functional domains in JRLs may help plants to adapt to the highly variable growth environment. The structural composition of JRL proteins varies greatly, but all of them contain a relatively conserved jacalin domain, which is generally composed of about 125 conserved amino acid residues, mainly including non-polar amino acids such as glycine and phenylalanine [7]. This sequence heterogeneity of JRL proteins strongly indicates their divergence in functional roles [6].

Since the first identification in the seeds of jackfruit (*Artocarpus integrifolia*) [8], JRLs have successively been found in more than ten plant species. At present, genome-wide identification of JRL proteins has been carried out in some plant species. There are at least 48 *AtJRLs* in *Arabidopsis thaliana* [9], 30 *OsJRLs* in rice (*Oryza sativa*) [2], 19 *ZmJRLs* in maize (*Zea mays*) [10], 65 *TaJRLs* in wheat (*Triticum aestivum*) [3], and 25 *PeJRLs* in moso bamboo (*Phyllostachys edulis*) [10]. However, genome-wide analysis of *HvJRLs* in barley is scarce, although barley is a major food and feed crop worldwide.

Several plant JRL genes have been functionally characterized in recent years. Some JRLs are associated with plant growth and development. In wheat, *TaVER2* regulates flowering and spikelet development, as it is induced during vernalization [11,12]. Overexpression of *OsJAC1*, a mannose-binding JRL, suppresses the coleoptile and stem elongation in rice [13]. In Arabidopsis, four antagonistic JRLs, namely, *JAL31*, *ATJAL23*, *PBP1/AtJAL30,* and *AtJAL22*, regulate the size of ER body-type b-glucosidase complexes [9], and *AtJAC1* functions in flowering time control [14]. Some JRLs were related to biotic stress responses [15,16,17,18]. The expression of *OsJRL* genes was induced by *Magnaporthe grisea* infection [19]. *Orysata* (it was also called *OsSalT* or *OsJRL*) is up-regulated under pathogen and insect attacks [20]. The above mentioned *OsJAC1* is involved in defense against pathogen attacks [21]. In addition, some JRLs play roles in response to abiotic stresses, such as salt [22,23], drought [24,25], and hormones [12,26,27]. In recent years, many studies have showed that the JRL family genes play important roles in salt stress response, including *Orysata* and *OsJRL40* in rice [28,29,30], *HvHorcH* in barley [31], a *PeJRL* in poplar (*Populus euphratica*) [32], and several *TaJRLs* in wheat [3]. Nitrogen deficiency is a common problem in agricultural production worldwide. However, no evidence of a role of JRLs in LNSR has been reported.

Despite the importance of plant JRLs, current knowledge about molecular characterization of *HvJRLs* in barley is far from sufficient. At present, only four *HvJRL* genes, namely, *HvJPR*, *Horcolin*, *HvLem2*, and *HvHorcH*, have been reported. They are mainly related to beta-glucosidase aggregation, jasmonic acid-inducible, salicylate-inducible, and salt stress responses, respectively [27,31,33,34,35]. So far, no studies of *HvJRLs* on nutritional stress responses have been reported. In the current study, we conducted a genome-wide identification and analysis of the *HvJRL* gene family in barley. Meanwhile, transcriptomic data, obtained from the two barley genotypes (XZ149, LN-tolerant, and XZ56, LN-sensitive) [36] treated with LN stress, were re-analyzed to identify DEGs encoding HvJRLs [37,38]. Finally, we examined the function of *HvHorcH* in LNSR. The major objectives were to characterize the *HvJRL* gene family, identify the *HvJRL* genes in response to LN stress, and explore the roles of one *HvJRL* gene in LNSR, thus providing valuable information for further research on the functions and regulatory mechanisms of HvJRL proteins, especially those proteins that may be useful for LN tolerance breeding.

## 2. Results

### 2.1. Identification of JRL Genes in Barley

A total of 147 HvJRL protein sequences were identified by searching against the barley genome with Hidden Markov Model (HMM) profiles of the jacalin domain (PF01419) using the HMMER3 program [39,40]. Meanwhile, 150 HvJRL protein sequences were obtained using 30 OsJRL protein sequences in rice [2] through the BlastP program [41]. After removing redundant and incomplete sequences, the jacalin domain was confirmed with NCBI CDD [42] and SMART [43]. Finally, 32 *HvJRL* genes were identified in barley (Table 1 and Table S1). These genes included the published barley JRL genes, namely, *HvJPR* (HORVU2Hr1G014020) [35], *HvLem2* (HORVU0Hr1G031210) [27], *Horcolin* (HORVU1Hr1G000160) [34], and *HvHorcH* (HORVU7Hr1G059330) [31]. The representative protein sequences of the 32 *HvJRLs* (Table S2) were analyzed by EXPASY [44]. HvJRL proteins were composed of 62 (HORVU5Hr1G119370) to 1376 (HORVU5Hr1G063690) amino acids, and their corresponding molecular weight (MW) ranged from 6.65 kDa (HORVU5Hr1G119370) to 153.70 kDa (HORVU5Hr1G063690) (Table 1). The theoretical isoelectric point (pI) was highly variable, ranging from 4.31 (HORVU3Hr1G031550) to 9.64 (HORVU4Hr1G023680) (Table 1). Most HvJRLs were predicted to localize to the cytoplasm (30/32) and nucleus (28/32), while some were localized to other locations such as chloroplasts, extracell, and mitochondria (Table S1) using WoLF PSORT [45].

### 2.2. Structural Conservation Analysis of the Jacalin Domains in Barley

**Figure 1:**
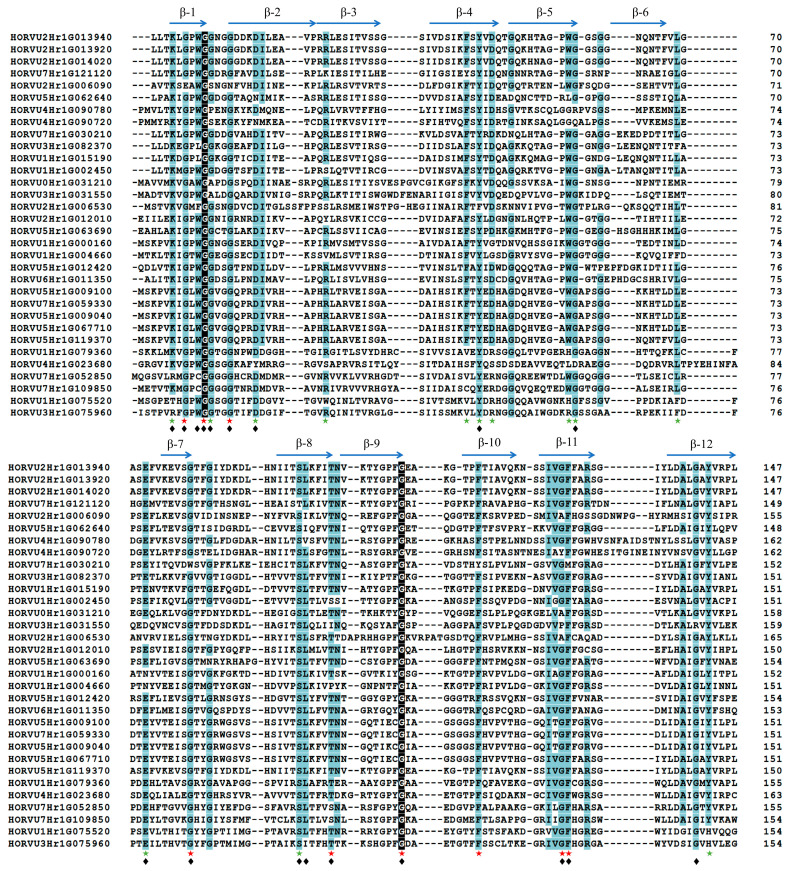
Multiple sequence alignment of the jacalin domains of HvJRLs. Amino acids with 100% identity are shown in black, and those with greater than 75% identity are shown in light blue. Dotted lines indicate gaps. Regions corresponding to β-strands are labeled by horizontal arrows above the sequences, and red stars indicate key residues in Heltuba used to package the three Greek key motifs. Green stars indicate residues that are highly conserved in HvJRLs but not present in Heltuba. Black rhombuses indicate highly conserved residues in both HvJRLs and PeJRLs.

To identify the conserved features of the jacalin domains in barley, a multiple sequence alignment was carried out based on their sequences in the HvJRL proteins. A total of 25 highly conserved amino acid residues (more than 75% identity) were identified in the jacalin domains of HvJRLs (Figure 1). The jacalin domain structure was well-established in a mannose-binding JRL protein Heltuba from *Helianthus tuberosus* [46], thus the identified conserved amino acid residues in HvJRLs were analyzed with Heltuba as a reference. There were 12 key residues necessary for the integrity of the β-prism fold in Heltuba, of which 9 are highly conserved in HvJRLs, while the Asn residue on β8 and Gly and Pro residues on β9 are less conserved in HvJRLs (Figure 1). In addition, 13 highly conserved residues, which were marked with green stars, were not present in Heltuba (Figure 1). And 18 conserved residues in the HvJRLs, which were marked with black rhombuses, were conserved in PeJRLs (Figure 1). Gly residue in β1 is invariant in all jacalin domains of HvJRLs, which was also observed in moso bamboo [10], indicating its importance for the biological function of plant JRLs.

### 2.3. Gene Structure, Conserved Motif, and Domain Analysis of HvJRLs

A phylogenetic tree was constructed on the 32 HvJRL proteins using the maximum likelihood (ML) method with 1000 bootstrap replicates. These proteins were classified into seven subfamilies (A–G) based on the clades with over 50% bootstrap support (Figure S1). To characterize the features of HvJRL proteins, the domain compositions based on the 32 HvJRL protein sequences were analyzed. The structural domains of HvJRL proteins are various, with different numbers of jacalin domains and different types of other domains (Figure S1). Of the 32 HvJRL proteins, 14 (43.75%) were merojacalins with only one jacalin domain, only 3 (9.38%) were holojacalins with multiple jacalin domains, and 15 (46.88%) were chimeric jacalins with jacalin domain(s) plus other domain(s) (Figure S1). For chimeric jacalins, 11 (73.33%) had an N-terminal dirigent domain plus one jacalin domain (Figure S1), and the remaining 4 had jacalin domain(s) plus other domain(s), including the PKc_like superfamily, B3, Motile_Sperm superfamily, NB-ARC superfamily, Rx_CC_like, LRR superfamily, and Rx_N (Figure S1). The HvJRL proteins in the same subfamily generally exhibited a similar domain composition. For instance, all the members in subfamily A, except HORVU7Hr1G030210, were composed of one dirigent domain and one jacalin domain, and all of those in subfamily F contained only one jacalin domain (Figure S1).

**Figure 2:**
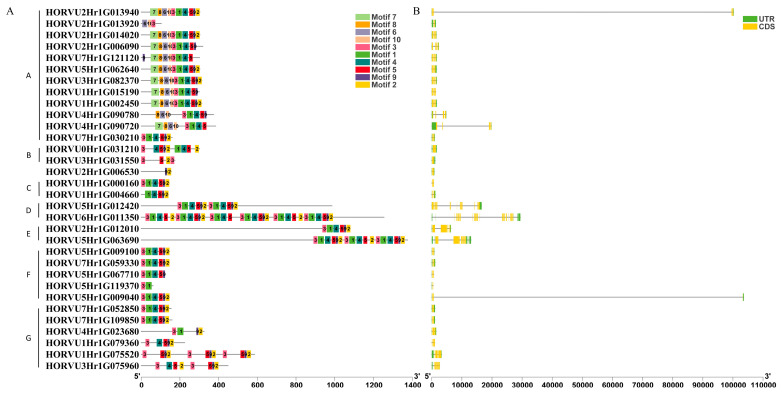
Phylogenetic tree, conserved motifs, and gene structure of the *HvJRL* genes from barley. (**A**) The motif compositions of 32 HvJRL proteins. The motifs, numbers 1–10, are displayed in different colored boxes. The sequence information for each motif is shown in Figure S2. The length of protein can be estimated using the scale at the bottom. The capital letters on the left of gene ID were the subfamilies to which the corresponding genes belong. (**B**) Exon/intron structure of *HvJRL* genes. Yellow boxes represent exons, and black lines represent introns. The upstream/downstream regions of *HvJRL* genes are indicated in green boxes. The length of exons can be inferred from the scale at the bottom.

In order to support the phylogenetic relationship, the gene structure analysis of *HvJRLs* was displayed. The result showed that most *HvJRLs* have 2–5 exons, with the exceptions of three chimeric lectins, including HORVU5Hr1G063690 (9), HORVU5Hr1G012420 (12), and HORVU6Hr1G011350 (21) (Figure 2B). In general, most members in the same subfamily exhibited a similar gene structure (Figure 2B). For instance, 11 of 12 genes in subfamily A had four exons.

In addition, ten conserved motifs were predicted in the HvJRL proteins (Figure S2). Almost all members of the *HvJRL* family contain motif 3. Motifs 1, 3, 4, and 5 together make up the jacalin domain (Figure 2A). HvJRL proteins consist of 2–32 motifs, and their motif compositions in the same subfamily were generally similar (Figure 2A). For example, motifs 1, 3, 4, 5, 6, 7, 8, and 9 existed in almost all members of subfamily A. Interestingly, some motifs were unique to one or more subfamilies. For instance, motifs 6, 7, 8, and 10 are specific to the members of subfamily A.

### 2.4. Chromosomal Distribution and Gene Duplication of HvJRL Genes

**Figure 3:**
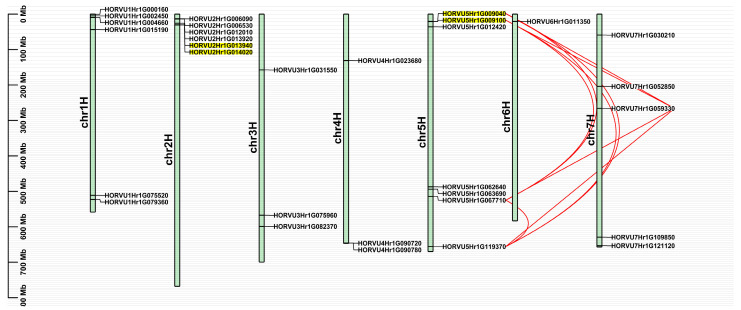
Chromosomal distribution and duplication events for JRL genes in barley. The tandem duplicated genes are highlighted in yellow, and the segmental duplicated genes are linked by red lines. The scale bar on the left indicates the length (Mb) of barley chromosomes.

The 32 *HvJRL* genes were unevenly distributed on the seven barley chromosomes, with most located at both ends of the chromosomes (Figure 3). More than 59% of the *HvJRL* genes were found on Chr5H (7), Chr1H (6), and Chr2H (6), and only one was located on Chr6H (Figure 3). In addition, HORVU0Hr1G031210 was distributed on scaffolds (Chr0H) (Table 1).

The duplication of *HvJRL* genes was determined using a Multiple Collinearity Scan toolkit (MCScanX) [47]. At least two tandem duplicated regions on Chr2H and Chr5H and nine segmental duplicated gene pairs on Chr5H and Chr7H were found in the *HvJRL* family (Figure 3 and Table S3). This result indicated that tandem and segmental duplication are important drivers for the generation of new *HvJRL* genes [10]. Moreover, each pair of duplicated genes belongs to the same subfamily, sharing the same domain composition and a similar motif composition and gene structure (Figure 2 and Figure S1).

### 2.5. Analysis of cis-Acting Elements in HvJRL Promoters

**Figure 4:**
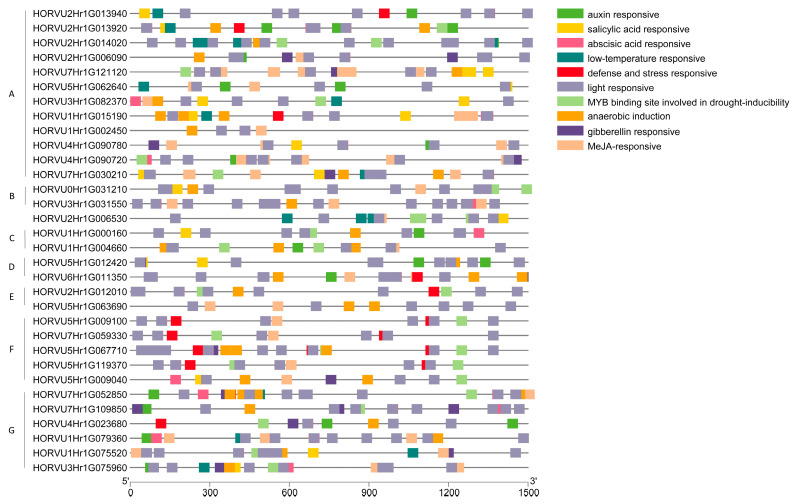
Predicted *cis*-elements in *HvJRL* promoters. Promoter sequences (−1500 bp) of 32 *HvJRL* genes were analyzed by PlantCARE. The upstream length to the translation starting site can be inferred according to the scale at the bottom. The capital letters on the left of gene ID were the subfamilies to which the corresponding genes belong.

*cis*-Acting elements are of importance for the regulation of gene expression. To analyze the potential regulatory mechanisms of JRL genes, the 1500 bp upstream promoter sequences from the start codon of *HvJRLs* were used to predict *cis*-acting elements through PlantCARE [48]. A large number of *cis*-elements were found in *HvJRL* promoters, which mainly contained hormone-responsive elements and stress-responsive elements (Figure 4, Table S4). For the hormone-responsive *cis*-acting elements, there were the MeJA response-related TGACG motif and the CGTCA motif; abscisic acid response-related ABRE; auxin response-related AuxRR-core and TGA-box; gibberellins response-related GARE motif, TATC-box, and P-box; coupled with the salicylic acid response-related TCA-element. The abiotic and biotic stress-responsive *cis*-acting elements included elements associated with low-temperature response LTR, anaerobic induction ARE, drought induction MBS, and defense and stress response TC-rich repeats (Figure 4, Table S4). The hormone- and abiotic stress-responsive *cis*-acting elements were present in almost all the *HvJRL* promoters, suggesting that *HvJRL* genes may play extensive roles in abiotic stresses and be involved in multiple plant hormone signaling pathways.

### 2.6. Evolutionary Analysis of JRL Gene Family

**Figure 5:**
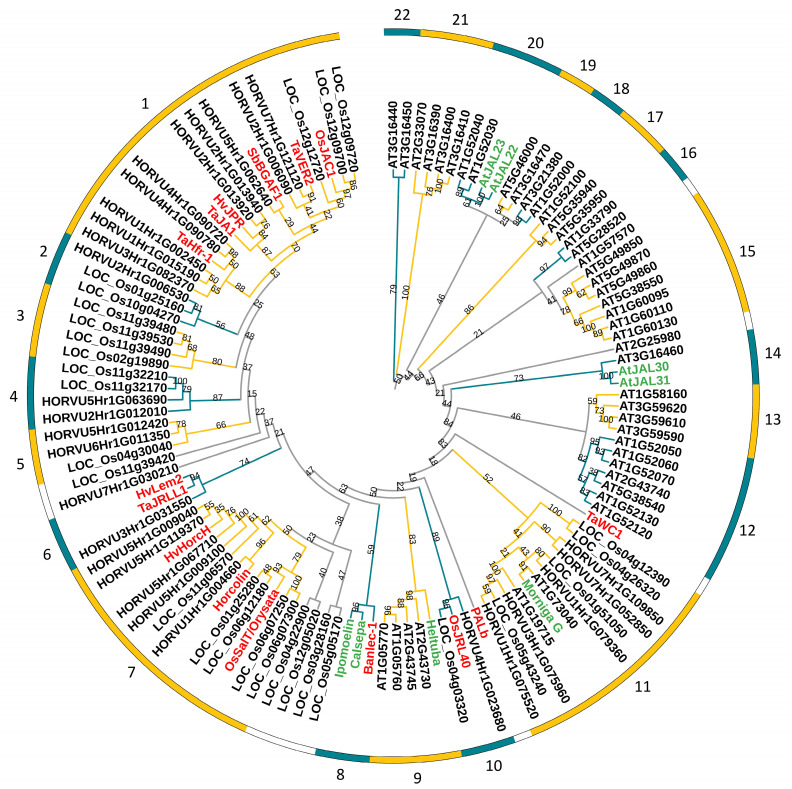
Phylogenetic analysis of JRL proteins among different species. A phylogenetic tree was constructed using MEGA v.10.1.8 with full-length amino acid sequences of JRLs from barley, Arabidopsis, rice, and those functionally known in other species, including TaWC1 (TAU32427), Moringa G (AY048576), PALb (AB099933), Heltuba (AF064029), Banlec-1 (AF001527), Calsepa (U56820), Ipomoelin (D89823), TaJRLL1 (HQ317136), TaJRLL1 (HQ317136), TaJA1 (AY372111), SbBGAF1 (ABI24164), and TaVER2 (BAA32786), and the number of bootstrap test replicates was set as 1000. Sequence accessions of functionally known proteins have been highlighted in red and green. The different-colored arcs indicate different subgroups of the JRLs. The numbers on the outside of the arc represent the subgroups to which the corresponding proteins belong.

To clarify the evolutionary relationships of JRL proteins between barley and other species, protein sequences from counterparts in barley, Arabidopsis, and rice, coupled with the verified functional JRLs in other plants, were used to construct the phylogenetic tree. A total of 48 *AtJRLs* (Table S5), which were also reported by Nagano et al. (2008) [9], were found by re-examining the Arabidopsis genome. All the 30 *OsJRLs* (Table S5) discovered by re-examining the rice genome were previously identified by Song et al. (2014) [3] and Han et al. (2018) [2], but LOC_Os01g25150 reported by Song et al. (2014) [3] has no JRL domain. Finally, an ML phylogenetic tree was constructed using protein sequences including 48 AtJRLs, 30 OsJRLs, 32 HvJRLs, and 12 JRLs in other plants (Figure 5). The results showed that the 122 genes could be grouped into 22 subgroups (subgroup 1~22). Notably, there were no HvJRLs in subgroups 9 and 12–22 but only JRLs from dicotyledonous plants, while subgroups 1–8 and 10 only contained JRLs from monocots, indicating that plant JRLs may have been functionally differentiated between monocots and dicots.

The potential functions of HvJRLs were predicted based on their functionally known homologs in the same subgroup (Figure 5). Numerous members in subgroups 1, 6, 7, 10, and 11 were involved in the response to biotic and abiotic stresses, such as pathogen resistance [16,21,49,50], response to hormones [26,27], salt tolerance [30,31,51], and drought tolerance [25]. The members of subgroup 1 might also be associated with beta-glucosidase aggregation [35,52], vernalization signaling, and spike development regulation [11,12]. The detailed functions of HvJRLs still need further study. The analysis has important reference value, although the evolutionary relationships could not be accurately deciphered for the functions of all the HvJRLs.

### 2.7. Expression Profiles of the HvJRLs in Response to Low-Nitrogen Stress

**Figure 6:**
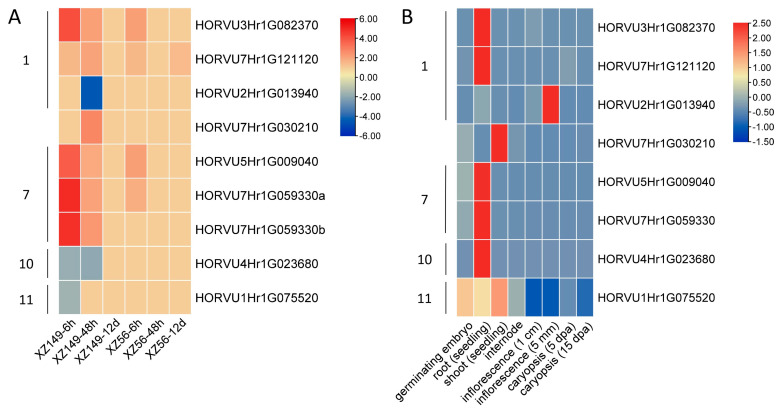
Expression profiles of the *HvJRL* DEGs. (**A**) Expression profiles of the *HvJRL* genes from the transcriptome in XZ149 and XZ56 at 6 h, 48 h, and 12 d under LN stress. Expression data were the ratio of FPKM of LN stress to that of the control at each time point. (**B**) Expression profiles (FPKM) of the *HvJRL* genes in different tissues of barley cultivar Morex. The color scale represents relative expression levels from high (red) to low (blue). The numbers on the left of each heat map are the subgroups to which the corresponding DEGs belong.

Many plant JRLs were confirmed to be involved in the biotic and abiotic stress responses. Combined with the phylogenetic and *cis*-element analyses, almost all the *HvJRLs* were potentially related to stress responses. However, it is unclear whether any *HvJRL* responds to nutritional stress. Thus, transcriptome data of barley roots under LN stress in our previous studies were employed and re-analyzed (Tables S6 and S7). Two barley genotypes (XZ149, LN-tolerant, and XZ56, LN-sensitive) were used for the RNA-seq analysis at 6 h, 48 h, and 12 d after LN stress [37,38]. Differentially expressed genes (DEGs) were screened with the threshold of FDR < 0.05 and FPKM ≥ 1 in at least one replicate. Finally, nine DEGs which encode eight HvJRL proteins were identified in the two genotypes (Figure 6A and Table S8). The RNA-Seq data were validated using nine *HvJRL* DEGs through real-time PCR analysis. The results showed that expressions of the identified *HvJRL* DEGs from real time PCR were highly consistent with that from RNA-Seq analysis (Figure S3).

Of the nine *HvJRL* DEGs, three were down-regulated and six up-regulated in their expression under LN stress. All the down-regulated DEGs were only changed in XZ149 but not changed in XZ56. All up-regulated DEGs except one were changed both at 6 h and 48 h in XZ149, but they only changed at 6 h or were not changed in XZ56. Interestingly, the fold change of these up-regulated DEGs was larger in the tolerant genotype XZ149 compared with the sensitive genotype XZ56 (Figure 6A). Noteworthily, only one DEG (HORVU7Hr1G121120, MLOC_34131) was found at 12 d after LN stress; it was up-regulated at 6 h and 48 h in XZ149 and at 6 h and 12 d in XZ56.

To further understand the tissue expression patterns of these *HvJRL* DEGs, the transcriptomic data in eight plant tissues of Morex were downloaded from the Expression Atlas database (https://www.ebi.ac.uk/gxa/plant/experiments (accessed on 21 September 2023)). A total of 30 *HvJRLs*, containing all the DEGs, were found expressed in the examined tissues from the available data (Table S9). And no DEG was expressed in all the eight tissues (Figure 6B), that is to say their expression was tissue-specific. Remarkably, all DEGs were expressed in roots, and four up-regulated DEGs showed the highest expression in roots among the eight tissues.

Generally, the DEGs in the same subgroup showed a similar expression pattern in their response to LN stress and in tissues, such as HORVU5Hr1G009040 and HORVU7Hr1G059330 in subgroup 7. However, there were also distinct expression patterns among the members of the same subgroup, for instance, HORVU2Hr1G013940 and HORVU3Hr1G082370 in subgroup 1, indicating that they may function in LNSR in different ways.

### 2.8. HvHorcH Enhanced Low-Nitrogen Tolerance in Transgenic Arabidopsis

To confirm the RNA-seq results and to determine the function of *HvJRLs* in LNSR, *HvHorcH* (HORVU7Hr1G059330) was taken as a candidate gene, as two DEGs (MLOC_76780 and MLOC_1563) were both aligned to this gene, and their fold changes of the up-expression were the highest in LN-tolerant genotype XZ149 under LN stress (Figure 6A). Firstly, the tissue and LN responsive expression patterns of *HvHorcH* were analyzed in XZ149 and XZ56. The real-time PCR result showed that the expression of *HvHorcH* was the highest in the root among the examined tissues for both the two genotypes (Figure S4), which was in accordance with that in Morex (Figure 6B). To investigate the dynamic expression of *HvHorcH* in response to LN stress, seven time points (1 h, 3 h, 6 h, 24 h, 2 d, 6 d, and 12 d) were set after LN treatment (Figure S5). For leaves, *HvHorcH* was up-regulated at all the time points in XZ149, while it was down-regulated at 1 h, 3 h, 6 h, 24 h, and 12 d in XZ56. For roots, *HvHorcH* was up-regulated from 3 h to 2 d in XZ149, while it was only up-regulated at 3 h and 6 h in XZ56. In general, *HvHorcH* was mainly expressed in roots, and its expression pattern under LN stress was different in XZ149 and XZ56.

**Figure 7:**
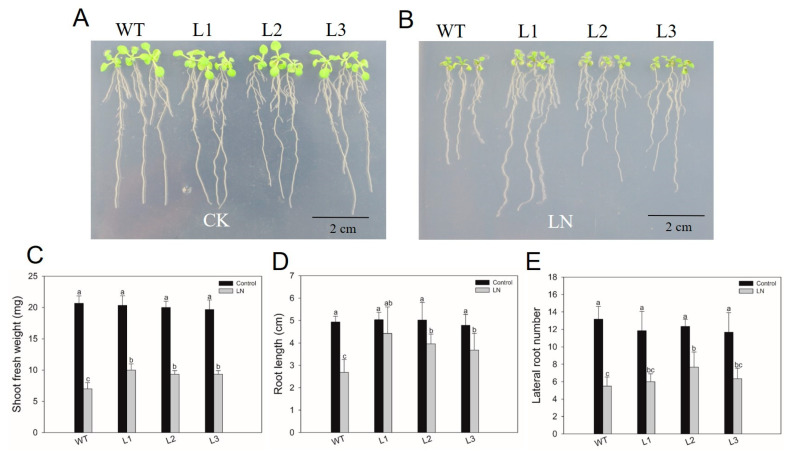
*HvHorcH* transgenic Arabidopsis showed enhanced low-nitrogen tolerance. The WT and three independent transgenic lines (L1, L2, and L3) were subjected to LN stress for 7 d and analyzed. (**A**,**B**) Morphology, (**C**) shoot fresh weight, (**D**) root length, (**E**) lateral root number. Four biological replications were performed. Different lowercase letters indicate a significant difference between treatments and genotypes at *p* < 0.05.

To further validate the function of *HvHorcH* in imparting LN tolerance, transgenic Arabidopsis plants overexpressing this gene were generated. Three independent transgenic lines, L1, L2, and L3, with high expression levels of *HvHorcH*, were used to evaluate the LN stress tolerance. The four-day-old of wild-type (WT) seedlings and three transgenic lines were treated for 7 d with a medium containing 0.1 mM NO_3_^−^ (10 mM NO_3_^−^ as a control) [53]. The growth performance of the three overexpressed lines was better than that of the WT, when exposed to LN stress, while there was no significant difference in the growth phenotype between the WT and transgenic lines in the control (Figure 7). In other words, all three lines displayed a significantly improved LN tolerance compared with the WT plants.

## 3. Discussion

JRL proteins are widely distributed in plants and are found in many species, including Arabidopsis, rice, wheat, maize, barley, *Sorghum bicolor,* and *Musa acuminata* [2]. Several of them have been demonstrated to be involved in plant growth regulation and stress responses [3,10,28,30,31,54]. Owing to their essential roles, the identification and characterization of JRL gene families and their expression profiles in response to abiotic stresses have been performed at the genome-wide level in several plant species, such as rice, wheat, and moso bamboo [2,3,10]. However, until now, no systematic studies have been reported on *HvJRLs* in barley nor on their roles in response to nutrition deficiency. Crops often suffer LN stress, as N deficiency is a common issue in agricultural production worldwide. Because JRL genes could take roles in so many environmental stress responses, such as salt, drought, and low-temperature stresses, we wondered if any of them respond to LN stress. In the present study, we performed a comprehensive investigation of *HvJRLs* and their expression under LN stress.

Here, a total of 32 *HvJRLs* were identified in barley combined from a BlastP and HMMER search (Table 1). Moreover, 48 *AtJRLs* and 30 *OsJRLs* were identified through a re-examined Arabidopsis and rice genome (Table S5). It has been reported that there were 46 to 123 JRL genes in cruciferous plants and 20 to 41 in graminaceous plants [2], which was accordance with our results. The accuracy of the *HvJRL* gene screening was further confirmed by multiple sequence alignment analysis, as all the 32 HvJRL proteins had jacalin domains (Figure 1). The HvJRL proteins have low sequence similarity, but the key sequences in their jacalin domain are relatively conserved, similar to those of Heltuba in *Helianthus tuberosus* and PeJRLs in moso bamboo [10,46]. According to the phylogenetic analysis, HvJRLs could be categorized into seven subfamilies, which was favored by the similar conserved domain and exon/intron structure in the same subfamily (Figure 2). Tandem duplication and segmental duplication were identified in the *HvJRL* family, and each duplicated pair existed in the same subfamily (Figure 2 and Figure 3), further confirming their similar evolutionary origins and biological functions. On the other hand, gene duplication acts as a primary driver allowing for sub-functionalization and neofunctionalization [55]. Herein, the duplicate genes may show different response patterns under LN stress, for example, HORVU2Hr1G013940 and HORVU2Hr1G014020, where one responds to LN stress and the other does not (Figure 6), indicating that functional divergence occurred as the *HvJRL* gene family expanded.

Phylogenetic relationships showed that most JRL genes from monocots and dicots have been grouped in some individual subgroups, indicating the basic characteristics’ divergency in this gene family between monocot and dicot plants. Interestingly, HvJRLs and OsJRLs are often clustered together, suggesting a closer relationship. The orthologous genes in the same subgroup often share similar functional roles. In this research, many HvJRL proteins were classified into some functional subgroups of functionally known JRLs, which provided a valuable reference for the study of the function of HvJRLs. Most functionally known JRLs in subgroup 1 are involved in agglutinating activity [35,50,52,56], which demonstrated that proteins from the same subgroup may display similar roles. Meanwhile, two members in subgroup 7, Orysata and HvHorcH, both confer a tolerance to salt stress [31,51], and the member OsJRL40 in subgroup 10 also positively regulated salt stress tolerance [30]. Both OsJAC1 and TaJA1 in subgroup 1 were associated with pathogen resistance [21,49,50], and TaJRLL1 in subgroup 6 was also involved in pathogen resistance [16]. These results suggest that there is functional redundancy in JRLs in different subfamilies. Therefore, the HvJRLs in subgroups 7 and 10 may play important roles in salt stress responses, while the HvJRLs in subgroups 1 and 6 participate in pathogen resistance. Notably, HvJRL proteins may serve similar functions in different ways. For instance, some *HvJRL* DEGs displayed diverse expression patterns under LN stress (Figure 6).

Members of the same subgroup also show multiple functions in their response to stress. For instance, in subgroup 8, Banlec-1 from *Musa acuminata* could prevent viral infection [57]; Calsepa in *Calystegia sepium* displayed agglutinating activity [56]; and Ipomoelin in *Ipomoea batatas* showed JA-mediated wound induction and antioxidation [58,59]. Additionally, some individual JRLs could display functions in multiple stress responses. For example, Orysata is induced by various stresses, including salt, drought, hormones, senescence, pathogen attack, and insect attack [20,23,28,60,61,62]. The diversity of multiple *cis*-elements in each *HvJRL* promoter may also indicate that one *HvJRL* has multiple functions in the complex environment. For example, Horcolin was related with agglutinating activity and jasmonic acid induction, and it also enabled the specific inhibition of cellular HIV infection [33,34,63].

Consequently, most *HvJRLs* were supposed to be involved in stress responses, such as salt tolerance in subgroup 7. However, no studies on the role of JRLs in nutrition deficiency has been published. To clarify whether JRLs participate in the LNSR, the transcript profiles of *HvJRLs* were analyzed employing two barley genotypes with differences in low-nitrogen tolerance. As a result, nine DEGs encoding eight HvJRL proteins were identified under LN stress (Figure 6A). In general, the fold change of six up-regulated DEGs was higher in XZ149 than that in XZ56, and the increase in the abundance of their transcripts lasted longer in XZ149 than in XZ56 (Figure 6A). Notably, MLOC_4840 (HORVU7Hr1G030210) and MLOC_1563 (*HvHorcH*) were up-regulated only in XZ149 (Figure 6A). Moreover, both MLOC_1563 and MLOC_76780 were sequenced to the *HvHorcH* gene. Thus, *HvHorcH* might play key roles in LNSR. To further reveal the function of *HvHorcH* in LNSR, the 35S:*HvHorcH* transgenic Arabidopsis was obtained. The transgenic Arabidopsis seedlings exhibited enhanced LN tolerance (Figure 7). The specific function requires further verification and discussion in the future.

## 4. Materials and Methods

### 4.1. Identification of JRL Genes in Barley

Genome data of Morex barley were downloaded from the Phytozome v.13 database (https://phytozome-next.jgi.doe.gov/ (accessed on 6 August 2023)). The Hidden Markov Model (HMM) profile of the jacalin-like lectin domain (PF01419) was obtained from the PFAM database (https://www.ebi.ac.uk/interpro/entry/pfam/#table (accessed on 10 August 2023)) [39]. The HvJRL proteins were identified from the barley protein database using the HMMER3 program with a threshold of e-value < 1 × 10^−20^ [40]. Meanwhile, 30 OsJRL proteins reported by Han et al. (2018) were used as query sequences to search for predicted HvJRL proteins with a threshold of e-value < 1 × 10^−10^ [41]. After eliminating redundant and incomplete protein sequences, the predicted HvJRL sequences were confirmed by the presence of the jacalin domain using NCBI-CDD (https://www.ncbi.nlm.nih.gov/cdd/ (accessed on 10 August 2023)) [42] and SMART (http://smart.embl-heidelberg.de/ (accessed on 10 August 2023)) [43]. Representative sequences of HvJRL proteins obtained by Tbtools v.1.120 [64] were taken for subsequent analysis. The molecular weight (MW), number of amino acids, and isoelectric point (pI) of HvJRL proteins were analyzed using ExPASy v.7 (https://web.expasy.org/protparam/ (accessed on 12 August 2023)) [44]. Subcellular localization predictions were generated through WoLF PSORT (https://wolfpsort.hgc.jp/ (accessed on 12 August 2023)) [45].

### 4.2. Sequence Alignment and Phylogenetic Analysis

The alignment of the core jacalin domain sequences of HvJRLs were carried out with ClustralW (http://www.genome.jp/tools/clustalw/ (accessed on 16 August 2023)) [65], and the conserved sequences were visualized with DNAMAN (v.2). Genome data of Arabidopsis and rice were retrieved from the Arabidopsis Information Resource database (http://www.arabidopsis.org (accessed on 6 August 2023)) and the Rice Genome Annotation Project (http://rice.uga.edu/ (accessed on 6 August 2023)), respectively. The AtJRLs in Arabidopsis and OsJRLs in rice were re-examined from the corresponding protein database employing the methods described above. Finally, 50 AtJRL and 30 OsJRL protein sequences were identified and used for phylogenetic analysis. An ML phylogenetic tree with 32 HvJRLs and a tree containing 112 JRL proteins from barley, Arabidopsis, rice, and other plants were constructed with 1000 bootstrap replicates in MEGA v.10.1.8, respectively.

### 4.3. Gene Structure, Conserved Motif, and Domain Analysis

The alignment of the cDNAs and their corresponding genomic DNA sequences of *HvJRLs* was performed to determine their exon/intron structure. The conserved motifs in HvJRLs were obtained using the MEME 5.5.1 online software (https://meme-suite.org/meme/ (accessed on 12 August 2023)) [66]. A visual map of gene structures and motif compositions was displayed through TBtools v.1.120 [64]. The conserved structural domains identified with the NCBI CDD and SMART were also visualized using TBtools v.1.120 [64].

### 4.4. Chromosomal Distribution and Duplication of HvJRL Genes

The chromosome location of *HvJRLs* was mapped based on their specific positions in the barley genome by TBtools v.1.120 [64]. The gene duplications of *HvJRLs* were determined by MCScanX (http://chibba.pgml.uga.edu/mcscan2/#tm (accessed on 17 August 2023)) [47]. If the alignment sequence covers more than 70% of the longer gene and the sequence identity is greater than 70%, it was defined as a gene duplication event [67]. The collinearity relationships were visualized using TBtools v.1.120 [64].

### 4.5. cis-Elements in the Promoter Regions of HvJRLs

To gain a preliminary understanding of the potential regulatory mechanism of the *HvJRL* genes, the 1500 bp upstream sequences from the start codon of the *HvJRLs* were used to determine the *cis*-elements’ distribution in PlantCare (http://bioinformatics.psb.ugent.be/webtools/plantcare/html/ (accessed on 16 August 2023)) [48]. The stress-related *cis*-elements in each promoter region were visualized using TBtools v.1.120 software [64].

### 4.6. Vector Construction and Arabidopsis Transformation

The full-length coding sequence of *HvHorcH* was cloned in LN-tolerant genotype XZ149. In brief, total RNA was extracted from the LN stress treated roots of XZ149. After quality monitoring, first-strand cDNA was synthesized with oligo dT primer and Random 6 mers (Takara, Kyoto, Japan). And then *HvHorcH* was cloned using its gene specific primer. It was then recombined into a pCAMBIA1300 vector with a flag tag. The recombinant 35S:*HvHorcH*-flag vector was used to transform *Agrobacterium tumefaciens* GV3101. And then the MES suspensions containing transformed Agrobacterium were transferred into the inflorescence of Arabidopsis (Col-0). The positive transgenic lines were screened through genomic DNA PCR with a *HvHorcH*-specific primer. The expression of the *HvHorcH* was analyzed by semiquantitative RT-PCR. The primers are listed in Table S10.

### 4.7. Plant Materials and Treatments

The transcriptome analysis was carried out using roots of XZ149 and XZ56. The barley seedlings were hydroponic with the basic culture solution containing 2 mM NaNO_3_ in a plant growth chamber (22/18 °C, day/night). Three-leaf-stage seedlings were treated with the nutrient solution containing 0.2 mM N (2 mM N as control) according to Yang et al. (2014) [68]. The culture medium was continuously aerated with an air pump and renewed every five days. The details were the same as those reported by Quan et al. (2016) [37]. The samples were taken at 6 h, 48 h, and 12 d under treatment. Finally, 24 samples (2 treatments (control and LN stress) × 3 time points (6 h, 48 h, and 12 d after LN stress) × 2 genotypes (XZ149 and XZ56) × 2 biological replications) were prepared for RNA-seq analysis. For real-time PCR analysis, we performed the same experiment as above, and then leaves and roots were taken 1 h, 3 h, 6 h, 24 h, 48 h, 6 d, and 12 d after LN stress.

The Arabidopsis genotypes used for phenotypic analysis under LN stress were the Col-0 ecotype (WT) and homozygous 35S:*HvHorcH* T3 transgenic Arabidopsis lines. The experiment was carried out in a plant growth chamber (25 °C) with a photoperiod of 16 h light/8 h dark and a light intensity of 100 µmol m^−2^ s^−1^. The Arabidopsis seeds were vernalized for three days and then cultivated for four days with basic medium in 13 cm × 13 cm vertical agar plates. The seedlings with uniform size were treated with the medium of 1 mM NO_3_^−^ (10 mM NO_3_^−^ as control). The basic medium was prepared according to Remans et al. (2006) [53] with a small modification, containing 10 mM KNO_3_, 2.5 mM MES, 1 mM KH_2_PO_4_, 0.5 mM CaSO_4_, 0.5 mM MgCl_2_, 50 µM NaFe(III)-EDTA, 50 µM H_3_BO_3_, 12 µM MnCl_2_, 1 µM ZnCl_2_, 1 µM CuCl_2_, and 0.03 µM (NH_4_)_6_Mo_7_O_24_. The K concentration was supplemented to 10 mM by adding KCl in the media for the LN treatment [69]. The growth phenotype was determined after 7 days under LN stress.

### 4.8. Transcriptome Analysis of HvJRL Genes in Response to LN Stress

A total of 24 cDNA libraries were constructed. Briefly, poly-A containing mRNA was obtained from the total RNA with poly-T oligo-attached magnetic beads. Following purification, the random fragmentation of mRNA was generated using divalent cations under elevated temperature and then reversely transcribed into cDNA. After adenylation of the 3′ ends of cDNA fragments, sequence adapters were ligated on both ends. The purified cDNA library fragments were selectively amplified and enriched. Then, the purified and quantified PCR products were applied for the cluster generation processing. Subsequently, each final library was sequenced on an Illumina NextSeq 500 platform to generate 2 × 75 bp pair-end reads.

After removing the adapter sequences, empty sequences, and low-quality bases from raw reads, quality analysis of the clean data was performed. And then, the clean reads were aligned to the Morex barley genomes using the ultra-high-throughput short read aligner on TopHat 2.1.1 (https://ccb.jhu.edu/software/tophat/index.shtml (accessed on 20 August 2023)), followed by the identification of splice junctions.

The abundance of the gene expression was calculated using FPKM (fragments per kilobase of exon per million mapped fragments) [70]. The differential expression of the transcripts under LN stress was re-analyzed employing DESeq2 [71]. The thresholds of FDR < 0.05 and FPKM ≥ 1 in at least one replicate were used to identify DEGs [72]. The DEGs were annotated using the genomes of Arabidopsis and Morex barely as reference. The fold change of *HvJRL* DEGs was calculated by the ratio of FPKM of LN stress/control and displayed in the heatmaps produced by TBtools v.1.120 software [64]. The datasets used are available on the following websites: https://doi.org/10.1186/s12870-016-0721-8 and https://doi.org/10.1186/s12870-019-1668-3 [37,38].

### 4.9. Tissue Expression Profile of JRL Gene Family in Barley

Transcriptomic data of Morex tissue were downloaded from the EMBL-EBI database (https://www.ebi.ac.uk/gxa/plant/experiments (accessed on 21 September 2023)) [73]. Eight different tissues included 4-day embryos dissected from germinating grains, shoots from seedlings (10 cm shoot stage), roots, developing inflorescence (5 mm and 1–1.5 cm), the third internode at the six-leaf visible stage, and 5 and 15 days post-anthesis developing grain. The tissue expression levels of *HvJRL* genes were obtained from the data and presented by heatmap.

### 4.10. Real-Time PCR

RNA of each sample was extracted with the FastPure Plant Total RNA Isolation Kit (Vazyme, Nanjing, China), followed by the first-strand cDNA synthesis using Hiscript III Reverse Transcriptase (Vazyme, Nanjing, China). The gene-specific primers were designed by primer-blast (https://www.ncbi.nlm.nih.gov/tools/primer-blast/ (accessed on 12 September 2023)), and *HvGAPDH* was used as the internal control gene to normalize all the data (Table S10). The real-time PCR was conducted in three biological replicates and three technical replicates on a CFX96 system (Bio-Rad, Hercules, CA, USA). The comparative CT method was used to calculate the relative expression which means that the fold change referred to the expression in the control [74].

### 4.11. Statistical Analysis

Significant differences for physiological traits were tested with Duncan’s Multiple Range Test on SPSS 16.0 software, and the difference at *p* < 0.05 was considered significant. The bar charts were generated in SigmaPlot 10.0.

## 5. Conclusions

In the present study, the barley JRL family genes were systematically characterized. The comparative transcriptome data indicated that at least eight *HvJRL* genes were associated with LNSR in barley. The expression pattern of *HvHorcH* under LN stress was further analyzed, and its potential functions in LNSR were also confirmed using the transgenic method. The *HvJRL* DEGs in XZ149 may be of significance for further functional study of the JRL genes in LNSR. This work may provide new candidate genes for breeding barley cultivars with LN tolerance.

## Figures and Tables

**Table 1:** Information on 32 *HvJRL* genes and their encoded proteins in barley.

Gene ID	Chromosome Location	Amino Acids	Molecular Weight (kDa)	pI
HORVU0Hr1G031210/LEM2	chrUn:186390238-186406458	302	32.10	5.78
HORVU1Hr1G000160/Horcolin	chr1H:475026-476135	146	15.05	7.95
HORVU1Hr1G002450	chr1H:4960525-4962190	314	33.16	6.94
HORVU1Hr1G004660	chr1H:9609641-9610836	142	14.94	4.95
HORVU1Hr1G015190	chr1H:43686713-43688076	301	32.66	5.57
HORVU1Hr1G075520	chr1H:511072663-511076165	586	63.44	6.64
HORVU1Hr1G079360	chr1H:522823378-522824959	208	22.34	9.22
HORVU2Hr1G006090	chr2H:12993110-12995463	319	35.34	6
HORVU2Hr1G006530	chr2H:13676587-13677516	156	16.89	9.34
HORVU2Hr1G012010	chr2H:25563354-25569794	1082	122.10	6.55
HORVU2Hr1G013920	chr2H:30286228-30287832	104	11.10	5.23
HORVU2Hr1G013940	chr2H:30372662-30472925	304	32.69	5.64
HORVU2Hr1G014020/HvJPR	chr2H:30577014-30745532	304	32.66	6.17
HORVU3Hr1G031550	chr3H:157387209-157388376	175	18.77	4.31
HORVU3Hr1G075960	chr3H:567163924-567167067	449	48.41	9.18
HORVU3Hr1G082370	chr3H:598849424-598851100	314	33.72	5.98
HORVU4Hr1G023680	chr4H:131046735-131048211	325	35.11	9.64
HORVU4Hr1G0907204	chr4H:645950575-645970388	385	41.87	8.25
HORVU4Hr1G090780	chr4H:645995092-646003566	374	40.25	9
HORVU5Hr1G009040	chr5H:20488797-20637689	147	15.55	6.42
HORVU5Hr1G009100	chr5H:20927441-21001231	147	15.64	6.21
HORVU5Hr1G012420	chr5H:35699858-35716432	986	109.11	6.15
HORVU5Hr1G062640	chr5H:487199094-487200722	304	33.06	5.24
HORVU5Hr1G063690	chr5H:493882556-493895687	1376	153.70	6.46
HORVU5Hr1G067710	chr5H:514246154-514246870	125	13.21	6.21
HORVU5Hr1G119370	chr5H:655562953-655564277	62	6.65	6.96
HORVU6Hr1G011350	chr6H:20611501-20640921	1254	134.57	6.75
HORVU7Hr1G030210	chr7H:59140290-59141279	160	17.25	5.03
HORVU7Hr1G052850	chr7H:203881126-203882208	155	16.63	6.5
HORVU7Hr1G059330/HvHorcH	chr7H:265851060-265852199	147	15.58	6.02
HORVU7Hr1G109850	chr7H:629167675-629168735	160	17.45	7.72
HORVU7Hr1G121120	chr7H:653151090-653152795	302	32.80	7.18

## Data Availability

The datasets used and/or analyzed during the current study are available from the first author on reasonable request, and her email address is bio_quanxy@ujn.edu.cn.

## Supplementary Materials

The supporting information can be downloaded at https://www.mdpi.com/article/10.3390/ijms242316641/s1.

## Abbreviations

DEG: differentially expressed gene; DPA, day post-anthesis; FPKM, fragments per kilobase of exon per million fragments mapped reads; LN, low nitrogen; LNSR, LN stress response; MCScanX, Multiple Collinearity Scan toolkit; ML, maximum likelihood; N, nitrogen.
