# Supplementary material for: Genome-Wide and Transcriptome Analysis of Jacalin-Related Lectin Genes in Barley and the Functional Characterization of HvHorcH in Low-Nitrogen Tolerance in Arabidopsis
Source: Int J Mol Sci. 2023 Nov 23;24(23):16641. doi: 10.3390/ijms242316641 (PMC10706597; doi:10.3390/ijms242316641)
Supplement: Supplementary file 1 [file ijms-24-16641-s001.zip › Figure S1-S5 caption.pdf]

Figure S1 Phylogenetic tree and domain composition of HvJRLs. (A) Phylogenetic tree of 32 HvJRL proteins. The maximum likelihood phylogenetic tree was constructed using MEGA v.10.1.8 with 1000 bootstrap replicates. (B) The domain composition of HvJRL proteins.

Figure S2 Conserved motifs of JRL domains in barley.

Figure S3 Real-time PCR validation of nine differentially expressed genes (DEGs). Transcript levels of nine DEGs and the corresponding expression data of RNA-Seq. The columns represent relative expression obtained by RNA-Seq, and solid lines represent relative expression obtained by real time PCR.

Figure S4 Expression analysis of the *HvHorcH* in tissues of XZ149 and XZ56.

Figure S5 Expression analysis of the *HvHorcH* in XZ149 and XZ56 at seven time points after LN stress. Expression data were the values of LN/control at each time point.
