# Supplementary figures and images for: Genome-Wide and Transcriptome Analysis of Jacalin-Related Lectin Genes in Barley and the Functional Characterization of HvHorcH in Low-Nitrogen Tolerance in Arabidopsis
Source: Int J Mol Sci. 2023 Nov 23;24(23):16641. doi: 10.3390/ijms242316641 (PMC10706597; doi:10.3390/ijms242316641)

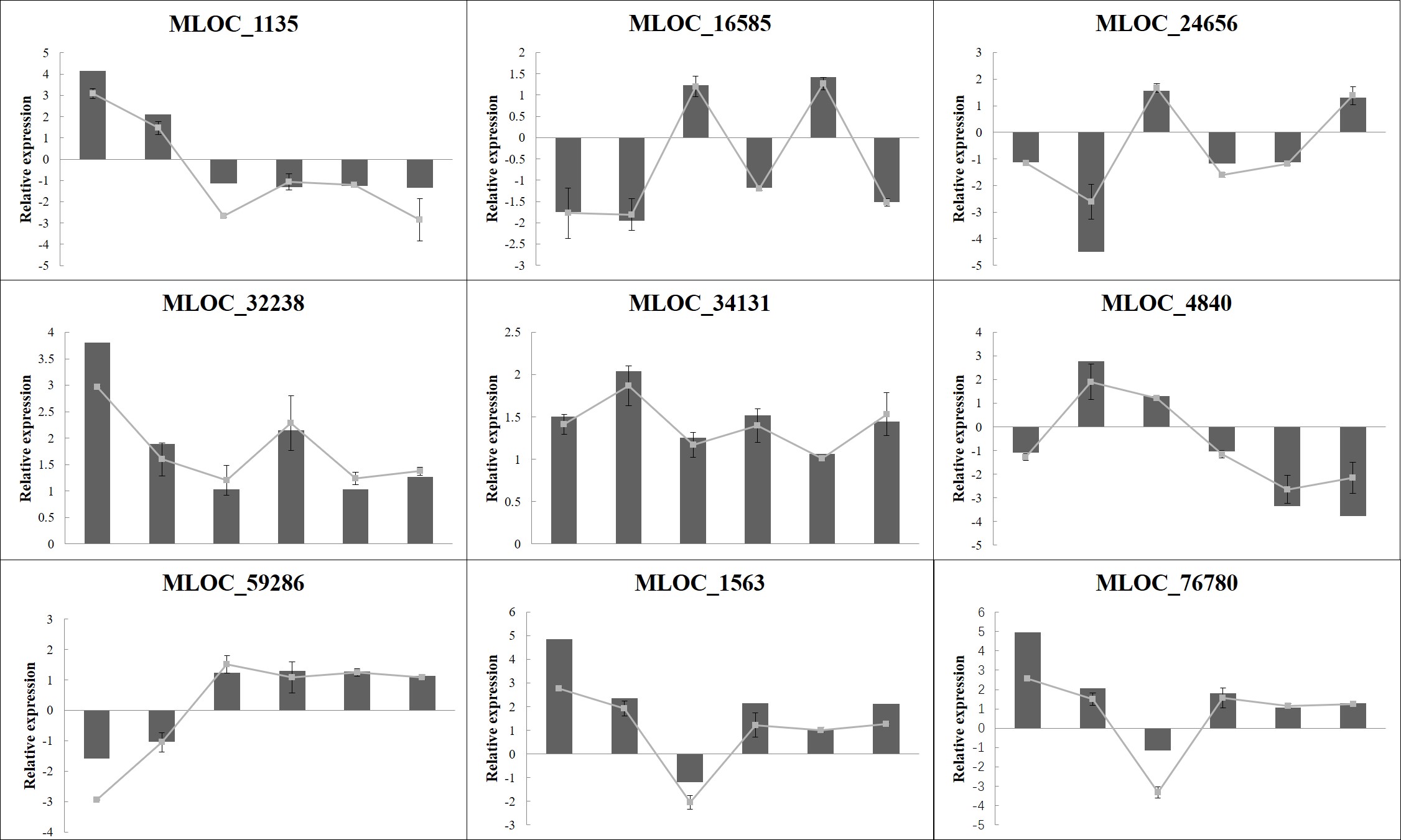

Supplement: Supplementary file 1 [file ijms-24-16641-s001.zip › Figure S3.jpg]

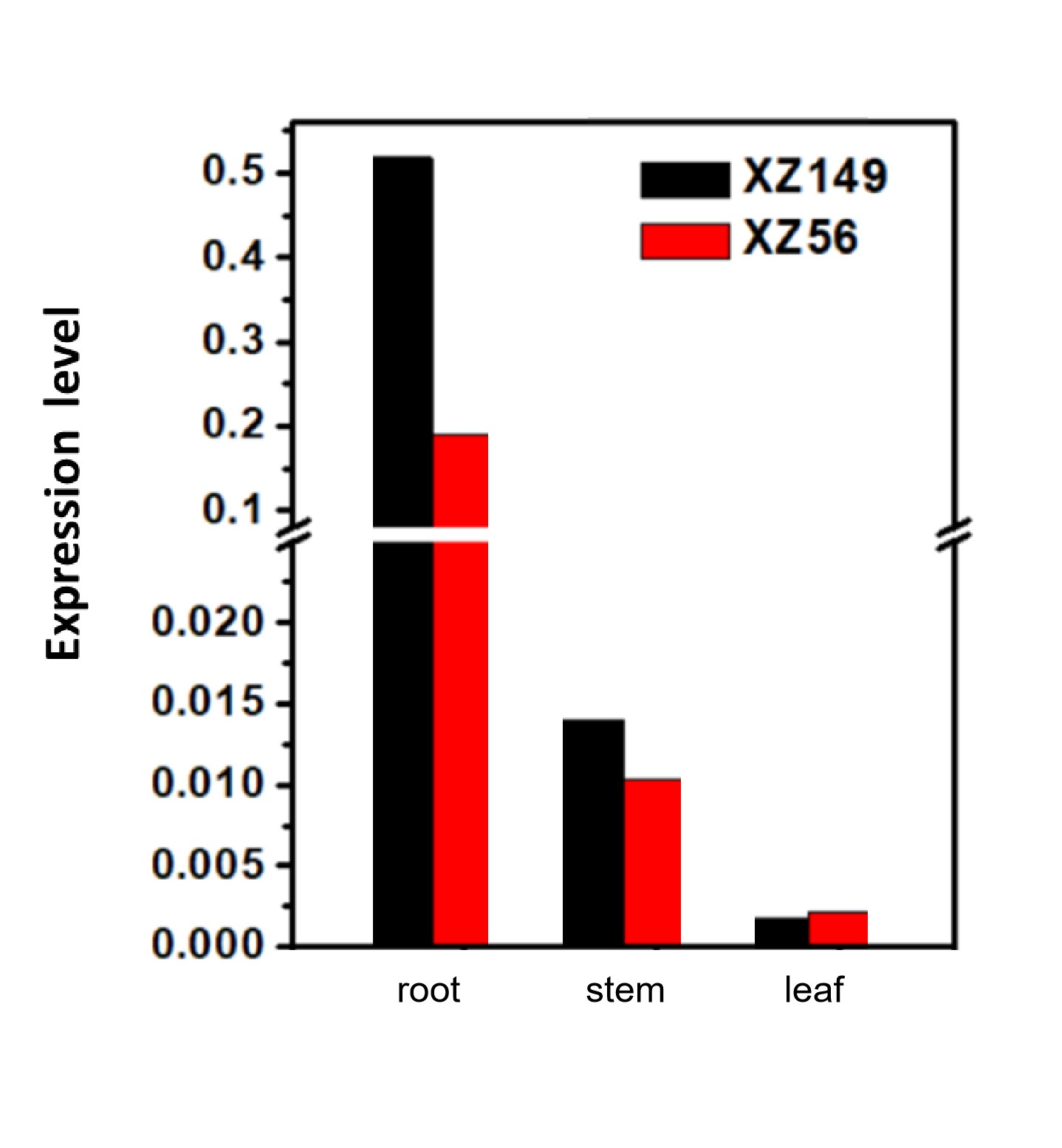

Supplement: Supplementary file 1 [file ijms-24-16641-s001.zip › Figure S4.jpg]

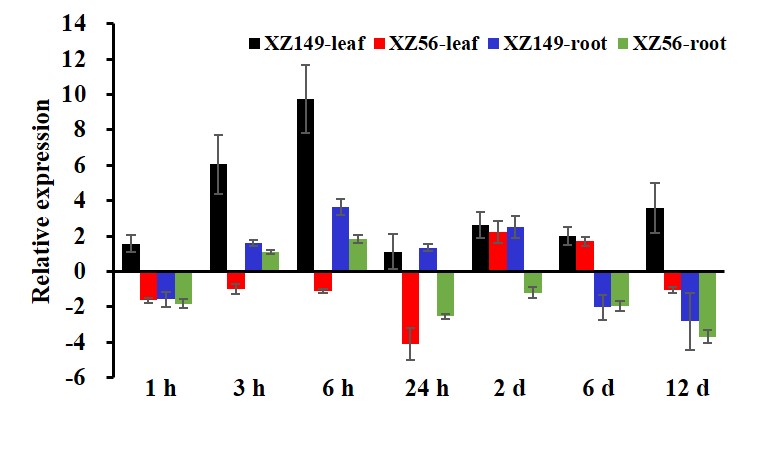

Supplement: Supplementary file 1 [file ijms-24-16641-s001.zip › Figure S5.jpg]
